# Preclinical Models of Bladder Cancer: Barrier, Metabolic, and Translational Susceptibility

**DOI:** 10.3390/ph19071116

**Published:** 2026-07-20

**Authors:** Tianjia Liu, Wei Li, Qinzhamusu Yin, Da Liu, Yong Wang, Ning Cui

**Affiliations:** 1 College of Pharmacy, Baicheng Medical College, Baicheng 137000, China; ltj@bcmc.edu.cn; 2 College of Pharmacy, Changchun University of Chinese Medicine, Changchun 130117, China; lw17638310029@163.com (W.L.); liuda@ccucm.edu.cn (D.L.); 3 Public Experimental Center, Changchun University of Chinese Medicine, Changchun 130117, China; 4 School of Clinical Medicine, Changchun University of Chinese Medicine, Changchun 130117, China; qinzhamusuyin@ccucm.edu.cn; 5 Northeast Asian Institute of Traditional Chinese Medicine, Changchun University of Chinese Medicine, Changchun 130117, China

**Keywords:** bladder cancer, preclinical models, urothelial barrier, susceptibility engineering, translational models

## Abstract

Preclinical bladder cancer models are often judged by tumor take, tumor growth or treatment inhibition, yet these endpoints do not reveal which bladder-specific constraints a given model preserves or bypasses. The bladder is shaped by a specialized urothelial barrier, urine exposure, cyclic filling and emptying, inflammatory injury, metabolic stress and intravesical treatment pressure. In this review, we use susceptibility engineering as an organizing framework for model selection and validation. We define susceptibility engineering as the deliberate definition, perturbation and reporting of model states that alter tumor initiation, adhesion, colonization, survival or therapeutic exposure. This framework groups cell lines, patient-derived organoids, cell-line-derived xenograft (CDX) and patient-derived xenograft (PDX) models, orthotopic transplantation, N-butyl-N-(4-hydroxybutyl) nitrosamine (BBN)-induced tumors, genetically engineered mouse models and large-animal platforms according to the biological constraints they test. We focus on three linked dimensions: urothelial barrier integrity and uroplakin-related tools; local colonization thresholds under bladder-specific selection; metabolic susceptibility involving peroxisome proliferator-activated receptor gamma (PPARG)-associated differentiation programs and candidate solute carrier family 25 (SLC25)-linked mitochondrial stress nodes. We further distinguish large-animal systems as platforms for local delivery, imaging, device testing and procedural scale rather than universal substitutes for mouse models. A susceptibility-based validation framework could improve model selection, explain divergent responses across systems and support tiered platforms that connect patient-derived biology, mechanistic mouse studies and clinically realistic intravesical evaluation.

## 1. Introduction

Bladder cancer is one of the most common malignancies of the urinary tract and is usually classified as non-muscle-invasive bladder cancer (NMIBC) or muscle-invasive bladder cancer (MIBC), two disease states that differ markedly in recurrence risk, progression patterns and treatment burden [1,2]. NMIBC often follows a prolonged relapsing course and is managed primarily by transurethral resection and intravesical therapy [3,4]. By contrast, MIBC is more prone to local progression and distant dissemination and often requires multimodal treatment, including chemotherapy, radiotherapy, immunotherapy and radical cystectomy, with substantial effects on quality of life [5,6]. Advances in molecular classification, immunotherapy, targeted therapy and patient-derived models have moved bladder cancer research towards more precise biological stratification and translational validation [7]. However, therapeutic progress has not resolved a more fundamental modeling problem: many animal systems still fail to reproduce tumor initiation, the intravesical environment, host barrier status and treatment response in a coordinated manner. This gap continues to limit the clinical translation of mechanistic discoveries and drug-screening studies.

Existing bladder cancer models provide complementary but incomplete views of the disease. Cell lines, patient-derived organoids (PDOs), cell-line-derived xenografts (CDXs), patient-derived xenografts (PDXs), orthotopic transplantation, N-butyl-N-(4-hydroxybutyl) nitrosamine (BBN)-induced tumors, genetically engineered mouse models (GEMMs) and large-animal platforms differ in tumor origin, implantation site, host background and translational scale [8]. These differences determine whether a model captures cell-intrinsic drug response, tumor initiation, local colonization, immune interactions or intravesical delivery. Model choice should therefore be guided by the biological threshold or therapeutic question under study, rather than by a simple hierarchy of experimental convenience.

Existing frameworks have classified bladder cancer models by induction method, histological similarity, immune background and therapeutic utility. These criteria remain necessary, but they do not fully explain why models with similar tumor cells can differ in engraftment, local growth or treatment response. We therefore propose susceptibility engineering as an additional organizing principle. Here, susceptibility refers to reportable host or model states that alter the threshold for adhesion, colonization, sustained growth or intravesical therapeutic exposure. This framing shifts model evaluation from asking whether a tumor forms to asking which biological constraints are preserved, intentionally perturbed or bypassed.

The urothelial barrier provides a first example of susceptibility. In the intact bladder, umbrella cells, with their tight junctions and apical uroplakin (UP) plaques, together with the extracellular glycosaminoglycan (GAG) layer, create a low-permeability surface that protects the tissue from urine and exogenous cells [9]. In orthotopic models, the same barrier limits tumor–cell adhesion and colonization, so pretreatments that improve engraftment can also introduce wound repair and inflammation. Barrier state should therefore be treated as a biological variable, not only as a technical obstacle.

However, barrier susceptibility is unlikely to act alone. Mitochondrial metabolism and oxidative stress can also shape the permissive state of host or tumor cells and may influence malignant transformation, colonization and treatment response [10,11].

After initial contact, tumor cells or mutated urothelial cells must survive urinary washout, oxidative stress, apoptosis, immune surveillance and treatment pressure before local growth becomes stable. Metabolic pathways may shape this permissive state. Mitochondrial transporters of the solute carrier family 25 (SLC25) family, including SLC25 member 21 (SLC25A21), illustrate how metabolite flux, reactive oxygen species (ROS) and apoptosis may influence bladder cancer growth, although this axis still requires broader in vivo validation [12,13].

Within this susceptibility-based framework, rabbit and other large-animal models are best viewed not as replacements for mouse models, but as intermediate platforms for procedural and translational validation.

Translational scale adds another layer to susceptibility-based model selection. Mouse models remain essential for genetic and mechanistic studies, but their small bladder volume limits repeated instillation, imaging follow-up, device testing and assessment of local drug exposure [14,15]. Larger-animal platforms can address these procedural questions more directly, especially when combined with patient-derived models or molecular subtype information [16,17]. Their role is therefore not to replace mice, but to connect mechanistic control with clinically realistic intravesical evaluation.

Against this background, this review uses susceptibility engineering to discuss how bladder cancer models can move from tumor-forming platforms towards systems guided by genetic information, local tissue context and route-specific therapeutic questions. We first outline the strengths and limitations of existing model platforms. We then examine the urothelial barrier as the first susceptibility gatekeeper in orthotopic modeling, because barrier integrity determines whether exogenous cells can attach to and persist within the bladder. Building on this barrier step, we distinguish early adhesion, local colonization and sustained-growth thresholds across PDO, xenograft, BBN and GEMM systems. We next discuss metabolic susceptibility, using peroxisome proliferator-activated receptor gamma (PPARG)-linked differentiation and SLC25A21 as bounded examples of stress-related model variables. Finally, we evaluate large-animal platforms as translational-scale systems and propose validation criteria for susceptibility-engineered bladder cancer models.

**Figure 1:**
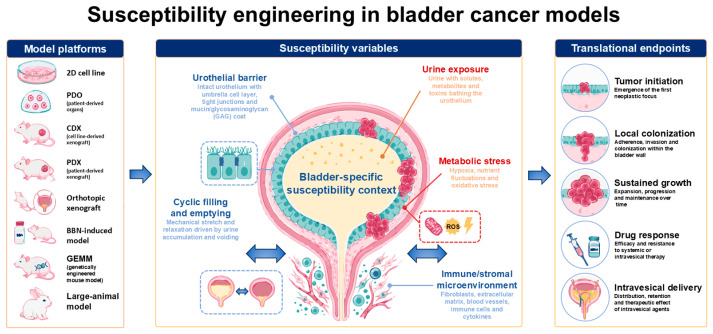
Susceptibility engineering links model choice to the biological constraints preserved, perturbed or bypassed by each platform. Barrier integrity, colonization threshold, metabolic stress and translational scale are treated as reportable model states that guide readout selection, therapeutic interpretation and tiered validation. Increased tumor take alone should be interpreted as empirical model optimization unless it is linked to initiation or colonization, the bladder microenvironment, molecular identity and route-relevant therapeutic evaluation.

A practical implication of this framework is that model improvement should not be judged by tumor take alone. A susceptibility-engineered model should connect tumor establishment or treatment response to four levels of evidence: the initiation or colonization threshold, the bladder microenvironment, molecular interpretability and route-relevant therapeutic evaluation. If an intervention increases tumor take without linking these levels, it is better described as empirical model optimization rather than susceptibility engineering. The overall framework of susceptibility engineering in bladder cancer models is summarized in Figure 1.

## 2. Types and Boundaries of Existing Bladder Cancer Models

Bladder cancer models are not a single experimental tool, but a spectrum of platforms that differ in complexity, application and biological fidelity. Commonly used systems include two-dimensional cell-line cultures, PDOs, CDXs, PDXs, orthotopic bladder xenografts, BBN-induced chemical carcinogenesis models, GEMMs and large-animal models [18]. These categories are not fully parallel. Subcutaneous and orthotopic transplantation describe the site of implantation, whereas CDX and PDX describe the source of tumor material [19,20]. Evaluation of bladder cancer models should therefore consider tumor origin, implantation site and host background together, and should compare controllability, modeling efficiency, tissue architecture, immune microenvironment, stromal components and suitability for therapeutic testing. This section summarizes the boundaries of these models and their value in basic and preclinical translational research.

Cell-line models remain useful because they provide controllable and reproducible systems for studying genetic alterations, pathway activity and drug response in bladder cancer [21,22]. Most are established by long-term culture of patient-derived tumor cells or by expansion in immunodeficient mice followed by re-isolation [23]. Their defined origin and simple handling make them practical for mechanism discovery and early pharmacological screening, although later validation is needed for anatomical and microenvironmental fidelity.

The main advantage of cell-line models is that they provide relatively stable platforms for fundamental tumor biology and preclinical pharmacology. For example, small-molecule fibroblast growth factor receptor (FGFR) inhibitors can suppress FGFR-dependent urothelial carcinoma growth in vitro and in vivo [24]. Urothelial carcinoma cell lines carrying fibroblast growth factor receptor 3 (FGFR3) mutations or FGFR3-transforming acidic coiled-coil containing protein 3 (FGFR3-TACC3) fusions can also serve as representative models for assessing sensitivity to selective FGFR inhibitors [25,26]. In addition, the co-expression extrapolation (COXEN) algorithm uses existing in vitro chemotherapy or radiosensitivity data to build multigene predictive models for malignant tumors, thereby supporting computational prediction of drug response [27]. These studies illustrate the continued practical value of cell lines in mechanism discovery, target validation and drug screening.

Bladder cancer cell lines can also be used to establish xenograft models. In a typical CDX model, established immortalized tumor cell lines are implanted into immunodeficient mice. Depending on the implantation site, CDXs can be further divided into subcutaneous and orthotopic bladder xenografts [28]. Subcutaneous CDXs form tumors rapidly, are technically simple and allow serial measurement of tumor volume, and are therefore commonly used for preliminary efficacy studies and tumor-growth experiments [29]. However, they are separated from the bladder anatomy and intravesical environment and cannot readily model urine exposure, bladder wall architecture, local stroma or intravesical treatment [30]. Because they usually require immunodeficient hosts, they also cannot fully capture the contribution of host immunity to tumor initiation, progression and treatment response [31]. Long-term passaged cell lines also fail to preserve the spatial organization and heterogeneity of patient tumors, further limiting their translational interpretation [23].

Orthotopic bladder transplantation models place tumor cells in the bladder lumen or wall, thereby preserving anatomical context more effectively than subcutaneous xenografts [32]. They are valuable for studying local colonization, invasion and intravesical therapy, but efficient implantation often requires perturbation of the mucosal barrier [33]. Thus, orthotopic models improve local relevance while introducing a key variable: how the barrier is altered to permit tumor–cell attachment.

Beyond cell lines and xenografts, PDOs provide a platform between two-dimensional culture and in vivo animal models. Organoids can be generated from patient tumor tissue, stem cells, organ-specific progenitors or dissociated tissues and are maintained in three-dimensional extracellular matrix-based culture [34]. Compared with conventional cell lines, PDOs can retain aspects of the histological architecture, molecular features and intratumor heterogeneity of the primary tumor, making them useful for patient stratification, drug-sensitivity testing and exploration of individualized treatment strategies [20,35]. As molecular classification and targeted therapy in bladder cancer continue to develop, PDOs provide an important bridge between clinical samples and functional validation [36,37]. Compared with xenografts, organoids require less tissue and less time to establish and are more amenable to in vitro genetic manipulation, including clustered regularly interspaced short palindromic repeats/CRISPR-associated protein 9 (CRISPR/Cas9)-mediated knockout of tumor-associated genes such as Trp53 and Stag2 [38]. However, PDOs remain simplified in vitro systems. They usually lack intact immune, vascular, neural and stromal components and cannot model urine exposure, intravesical pressure changes or drug retention within the bladder [39,40]. Their strengths therefore lie mainly in high-throughput, expandable and patient-specific functional testing, whereas interpretation of tumor microenvironment and local treatment response still requires validation in animal models.

Unlike cell-line-derived xenografts, PDX models are usually generated by directly implanting fresh patient tumor tissue into immunodeficient mice, thereby better preserving tumor morphology, genetic background and part of the clonal heterogeneity of the original tumor [41]. PDX describes the source of the tumor material rather than the implantation site, and PDXs can be established subcutaneously or further adapted to orthotopic implantation [19,42]. Studies have shown that bladder cancer PDXs can maintain considerable morphological and genomic concordance with the original patient tumors and can be used to evaluate chemotherapy, targeted therapy and the evolution of resistance. Responses to cisplatin–gemcitabine and FGFR-activated, human epidermal growth factor receptor 2 (HER2)-activated, phosphoinositide 3-kinase (PI3K)/mechanistic target of rapamycin (mTOR)-activated and mitogen-activated protein kinase kinase (MEK)-directed therapies, for example, indicate that PDXs provide a more patient-relevant validation platform for molecularly guided treatment. Orthotopic PDX or PDO-derived xenograft models can further place patient-derived tumors within the bladder environment, allowing assessment of tumor growth, metastatic propensity and local treatment response, thereby complementing subcutaneous models in anatomical and microenvironmental relevance [43]. Nevertheless, PDXs are not complete models of bladder carcinogenesis. Engraftment efficiency depends on sample quality and passage history, and clonal selection or marker drift can occur during passaging. Most PDXs also rely on immunodeficient hosts, limiting interpretation of immune microenvironment and immunotherapy response, a limitation only partly addressed by humanized systems [44]. PDXs are therefore best viewed as patient-relevant preclinical validation platforms rather than replacements for cell lines, BBN models or GEMMs. Their greatest value lies in assessing drug response, exploring resistance mechanisms and supporting precision-therapy studies while retaining patient-derived heterogeneity.

In contrast to transplantation models, BBN-induced chemical carcinogenesis is a non-transplant, orthotopic-like model of tumor initiation [9]. BBN is a nitrosamine carcinogen commonly used to mimic urothelial carcinogenesis associated with environmental carcinogen exposure [45]. BBN and its metabolites can induce urothelial tumor initiation and progression in immunocompetent animals [22]. The carcinogen is usually administered to mice or rats in drinking water, metabolized in vivo and then selectively delivered to the urothelium, where it drives a continuum from urothelial hyperplasia and carcinoma in situ (CIS) to invasive disease [46]. Importantly, BBN induction is not a fixed process, but is strongly shaped by genetic background. C57BL/6 is the most commonly used mouse strain, partly because its genetic and immune phenotypes are well characterized. Administration of 0.05% BBN in drinking water for 16 to 20 weeks induces urothelial carcinoma in most animals and is often used as a standardized protocol. However, strain-specific differences in BBN sensitivity, tumor latency and histopathology are substantial. ICR mice can develop CIS or invasive carcinoma earlier at similar concentrations, whereas BALB/c mice generally require longer induction to reach high tumor incidence [9]. FVB mice appear more prone to rapid invasive disease and may show glandular differentiation, a phenotype uncommon in C57BL/6 mice [47]. NON/Shi mice can display stronger local invasion, ureteral obstruction and metastatic tendency, but their tumor spectrum includes a higher proportion of renal pelvic lesions, suggesting that this strain does not purely model bladder urothelial carcinoma progression [48]. By contrast, conventional BBN-treated C57BL/6 mice rarely develop distant metastases unless observation is prolonged and endpoints are carefully monitored [9]. These differences indicate that BBN is not a single chemical bladder cancer model, but a modeling system shaped jointly by host genetics, dosing regimen and observation window.

Similar variation is seen in rat models, although their overall pathological spectrum differs from that of mice [49]. Wistar, Sprague Dawley and F344 rats can all develop urothelial injury or tumors after BBN exposure, but the lesions are often papillary, low-grade or non-invasive, and the incidence and stability of invasive disease are generally lower than in mouse models [50]. BBN-induced rat tumors may therefore be more suitable for studying papillary tumor formation, early urothelial injury or NMIBC-related questions, whereas mouse BBN models are more often used to model high-grade and invasive bladder cancer. Overall, BBN models are frequently used to study bladder carcinogenesis, progression mechanisms, immune microenvironmental changes and therapeutic intervention [51]. Their limitations include long induction periods and dependence of tumor timing and pathology on dose, duration, strain, sex and metabolism. Although chemically induced lesions mimic some features of human bladder cancer, they do not necessarily correspond to a defined patient subtype or driver genotype [52]. BBN models are therefore best suited to questions about how tumors arise and evolve in an intact host environment.

GEMMs complement transplantation and BBN models from a different direction. By activating oncogenes or inactivating tumor suppressor genes specifically in the urothelium, GEMMs allow candidate driver events, cells of origin, molecular subtypes and mechanisms of progression from NMIBC to MIBC to be examined in defined genetic contexts. Their major advantage is that tumors arise within immunocompetent hosts, making them well suited to causal analysis of the relationship between specific genes or signaling pathways and tumor phenotype [53].

However, bladder cancer GEMMs remain difficult to build. Bladder-specific promoters vary in strength, specificity and inducibility, and the urothelium is relatively resistant to invasive transformation. Many single oncogene or tumor-suppressor manipulations produce hyperplasia, CIS or non-invasive lesions rather than reproducible MIBC [54,55]. GEMMs are therefore most useful as causal platforms for defined genetic events and lineage questions, while detailed pathway cooperation is considered below in the context of sustained-growth thresholds.

Large-animal models are less common in bladder cancer research but useful for questions that require clinical operative scale [56]. Rabbit, porcine and canine systems can support intravesical instillation, local delivery, imaging follow-up, device testing and safety assessment more realistically than mouse models [17]. Their limitations include cost, ethical constraints, low throughput and limited genetic tractability. Detailed evidence for rabbit VX2, porcine and canine platforms is discussed in Section 6.

In summary, PDOs, PDXs, BBN models, GEMMs and large-animal models each compensate for different limitations of conventional cell lines and CDXs. PDOs and PDXs emphasize patient-derived features and individualized drug responses. BBN models and GEMMs are better suited to studying tumor initiation, progression and genetic drivers in intact hosts. Large-animal models mainly support local therapy and device translation. Model choice should not be guided by the search for a single optimal platform, but by the research question. Mechanistic studies require controllable genetic or chemical induction systems; drug screening requires high-throughput and reproducible platforms; and intravesical therapy evaluation requires orthotopic or large-animal models that preserve the bladder anatomy and delivery context. The major features, strengths and limitations of these model platforms are summarized in Table 1.

## 3. The Urothelial Barrier as a Gatekeeper in Orthotopic Bladder Cancer Modeling

From the bladder lumen outward, the urothelium consists of umbrella cells, intermediate cells and basal cells [60]. Small cuboidal basal cells and some intermediate cells express p63 and high-molecular-weight cytokeratins, although expression is lower in intermediate cells, probably reflecting their transitional state towards umbrella cell differentiation. Umbrella cells are large and often multinucleated. Their apical membrane contains a highly specialized, insoluble and polarized structure, the asymmetric unit membrane (AUM) [61]. The AUM consists of an outer leaflet facing the urinary lumen and an inner leaflet facing the cytoplasm, with the outer leaflet approximately twice as thick as the inner leaflet. The AUM is composed mainly of UPs and restricts urine reabsorption while maintaining the luminal barrier [62]. Umbrella cells are therefore not merely superficial cells in morphological terms, but key effectors of urothelial barrier function.

UPs are core components of the apical membrane of umbrella cells and include UPK1A, UPK1B, UPK2, UPK3A and UPK3B. They are highly enriched in umbrella cells and are absent or expressed only at low levels in basal and intermediate cells [63]. After synthesis, UPs enter the endoplasmic reticulum, but they do not function as isolated proteins. Instead, they first form dimers in specific combinations. UPK1A-UPK2 and UPK1B-UPK3A are the most common pairs, and a smaller amount of UPK1B-UPK3B pairing has also been reported [64]. Successful pairing is required for UPs to exit the endoplasmic reticulum and enter the Golgi apparatus [65]. In the Golgi, UPs are processed at N-glycosylation sites to form complex glycan structures, and these glycosylation events partly contribute to the asymmetric morphology of the AUM. Processed dimers further assemble into heterotetramers, with UPK3B-containing complexes often considered alternative heterotetramers [66]. Six heterotetramers have been reported to assemble into an approximately 16 nm uroplakin particle, and these particles then organize into semi-crystalline urothelial plaques, generating a low-permeability and low-adhesion luminal barrier [67].

This structural specialization gives the urothelial barrier a dual role in orthotopic bladder cancer modeling. As noted above, orthotopic xenograft models often require acid-base treatment, mechanical injury or electrocautery of the bladder epithelium because the normal urothelium resists exogenous tumor–cell adhesion. This resistance is mediated by the barrier formed by the apical membrane of umbrella cells, UP plaques, tight junctions and the GAG layer [68]. UP exocytosis can occur during bladder filling or when the tissue needs to resist entry of exogenous material. UPs are subsequently degraded and recycled through pathways that include lysosomes rather than simply returning to apical discoidal or fusiform vesicles. From a modeling perspective, the same barrier is both a protective mechanism for bladder homeostasis and the first threshold that exogenous tumor cells must cross to adhere and colonize the bladder lumen.

UP dysfunction or abnormal UP expression is generally thought to weaken the apical urothelial barrier and thereby lower the threshold for exogenous tumor–cell adhesion or orthotopic implantation. However, the relationship between UP alterations and bladder cancer is not a simple barrier-loss model. One study showed that antisense oligonucleotide-mediated inhibition of UPK1A suppresses proliferation and promotes apoptosis in T24 bladder cancer cells [69]. Another study using UPK1B siRNA reported reduced tumor–cell proliferation, migration and invasion accompanied by inhibition of Wnt/beta-catenin signaling [70]. These observations seem partly inconsistent with the role of UPK1B as a structural barrier component, but they suggest that some UPs may also regulate tumor–cell phenotypes. UP expression also reflects the state of the urothelial differentiation program. High UP expression indicates retention of mature urothelial or umbrella-like identity, whereas loss of UPs often suggests a shift towards a basal-like state with greater invasive potential. UPs are therefore important markers of luminal or umbrella-like differentiation, but luminal–basal classification also involves KRT20, GATA3, FOXA1, PPARG, KRT5/14 and p63, and should not be inferred from UPs alone [71,72].

Beyond their barrier and differentiation marker functions, UP-related genes provide urothelium-specific transcriptional elements for model construction. The earliest evidence came from studies of the UPK2 promoter. Investigators isolated an approximately 3.6-kb regulatory region upstream of the UPK2 transcription start site and used a LacZ reporter system to show that transgene expression was mainly localized to intermediate and umbrella-differentiated cells of the urothelium in transgenic mice, rather than being broadly expressed throughout the body [73,74]. This work provided an important genetic entry point for subsequent bladder cancer modeling. These promoter systems are relevant because they convert urothelial differentiation biology into model-engineering tools. UPK2 regulatory elements have been used to restrict transgene or viral expression to the urothelium, including oncolytic adenoviral and suicide-gene strategies [75,76,77]. The therapeutic designs differ across vectors, but the modeling implication is shared: UPK promoters provide a urothelium-specific entry point that may allow susceptibility states to be manipulated more reproducibly than by mechanical injury alone.

**Figure 2:**
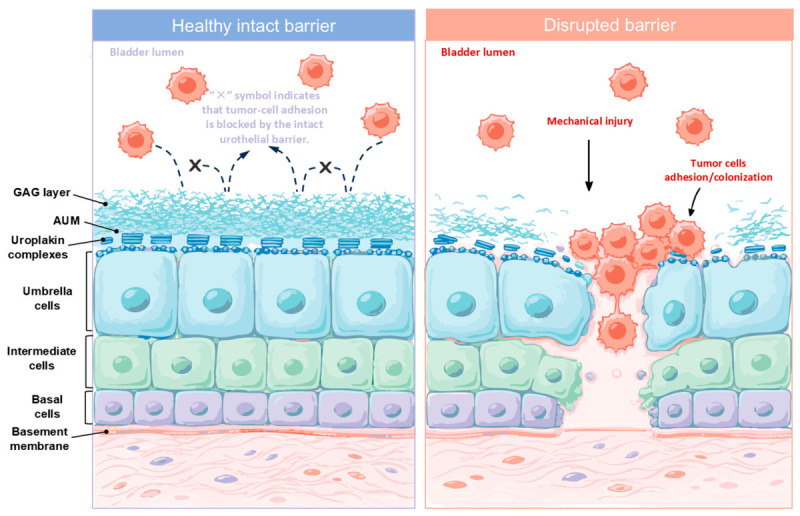
The urothelial barrier controls tumor–cell adhesion and colonization through umbrella cell differentiation, UP plaques, the GAG layer, tight junctions and injury-induced access to the basement membrane.

Thus, the link between UPs and bladder cancer models extends beyond barrier architecture. UP expression reports urothelial differentiation and tumor-phenotype changes, whereas UPK promoters provide mature tools for urothelium-specific genetic manipulation. Integrating these two layers may offer a route to more stable, controllable and intravesically relevant orthotopic susceptibility models. This barrier-dependent adhesion process is illustrated in Figure 2.

## 4. Local Colonization Thresholds: From Cell Survival to Sustained Growth

Tumorigenicity in bladder cancer models should not be reduced to whether a visible mass forms. Different models place tumor cells, or injured urothelial cells, under distinct selective pressures, and therefore require them to cross different biological thresholds. Cell lines and PDOs mainly test proliferation, clonal competition and drug response under artificial culture conditions. Orthotopic transplantation further requires exogenous tumor cells to contact, adhere within and resist washout from the bladder lumen. BBN-induced models and GEMMs do not involve exogenous cell implantation, but injured or mutant urothelial cells must still evade apoptosis, immune elimination and tissue-homeostatic constraints to form detectable clonal lesions. The thresholds discussed in this section are therefore not limited to intravesical adhesion, but encompass the continuous transition from transient survival to sustained local growth.

PDOs and xenografts test different parts of the colonization continuum. PDOs bypass the bladder lumen but expose patient-derived cells to matrix, growth-factor, passage and drug-selection pressures [20,35]. Subcutaneous xenografts test in vivo growth and vascularization, but bypass urothelial adhesion, urine exposure and intravesical drug dwell. Orthotopic CDXs, PDXs and PDO-derived xenografts add the local barrier and washout steps, but often require pretreatment, making the induced injury itself part of the model.

BBN-induced carcinogenesis reframes this threshold in a non-transplant setting. Rather than testing adhesion of exogenous cells, BBN models test whether injured urothelial clones can survive carcinogen exposure, inflammation and tissue-homeostatic pressure long enough to progress [22,78]. Because BBN must be metabolized and excreted in urine, the effective pressure on the urothelium varies with strain, dose, sex and exposure window [46]. The key point for susceptibility modeling is therefore not only whether tumors form, but which host and exposure conditions allow damaged clones to move from transient injury to stable expansion.

GEMMs further show that genetic alteration alone is not always sufficient to cross the thresholds for sustained growth and invasive progression. Kobayashi and colleagues noted that although GEMMs are well suited to studying candidate drivers in defined genetic backgrounds, relatively few bladder cancer mouse models reproducibly generate muscle-invasive or metastatic phenotypes. The p53/Rb axis provides one example. He and colleagues found that Rb1 loss alone does not directly induce bladder cancer, but instead activates an E2F1-p53 stress response, together with compensatory upregulation of Rb-family members such as p107, leading to cell-cycle arrest and apoptosis. Even combined Rb1 and Trp53 loss mainly produced delayed hyperplasia, nuclear atypia or rare superficial papillary lesions. Invasive lesions resembling human muscle-invasive urothelial carcinoma were more likely only when subthreshold BBN exposure was added to the Rb1/Trp53-deficient background [79]. This model indicates that cell-cycle deregulation, impaired DNA-damage response and carcinogen-driven selection pressure must act together before abnormal urothelial cells can more readily overcome progression barriers.

Similar principles apply to the PI3K/phosphatase and tensin homolog (PTEN)/mTOR, Wnt and rat sarcoma viral oncogene homolog/mitogen-activated protein kinase (RAS/MAPK) pathways. Puzio-Kuter and colleagues delivered adenovirus-expressing Cre recombinase (Adeno-Cre) intravesically to delete Trp53 and Pten simultaneously in adult mouse urothelium and found that combined loss induced invasive bladder cancer, whereas alteration of either gene alone was insufficient to produce the same phenotype. Rapamycin blocked tumor formation in this model, indicating that the synergy depends at least in part on mTOR signaling [80]. Ahmad and colleagues approached the question through the Wnt pathway and found that urothelium-specific beta-catenin activation caused only focal hyperplasia and was accompanied by PTEN upregulation. When beta-catenin activation was combined with Pten loss, the model formed papillary urothelial carcinoma with enhanced protein kinase B (AKT)/mTOR signaling [81]. Ras models are also dose- and combination-dependent: low-level activated Ha-ras usually causes simple hyperplasia, whereas stronger Ras signaling can induce low-grade non-invasive papillary tumors. When activated Ras is combined with Trp53 loss or beta-catenin activation, CIS, basal or squamous-like muscle-invasive lesions and epithelial–mesenchymal transition (EMT) or stemness-associated programs are more likely to emerge [55]. Thus, proliferative signaling does not automatically produce invasive progression. Entry into the MIBC state depends on whether p53-p21, PTEN/mTOR, Wnt or other homeostatic defense axes have also been lifted.

The Notch pathway illustrates the same principle from the perspective of differentiation maintenance. Maraver and colleagues found that NOTCH1 and NOTCH2 mutations in human bladder cancer reduce Notch activity. In mice, intravesical Adeno-Cre-mediated deletion of Psen1/Psen2 or Rbpj combined with BBN exposure shortened survival, accelerated tumor formation and promoted more invasive lesions with squamous differentiation [82]. Mechanistically, reduced Notch-HES1 activity was accompanied by decreased E-cadherin, increased vimentin and enhanced EMT features, suggesting that Notch may restrict urothelial tumor progression by maintaining epithelial identity and suppressing mesenchymal transition [83].

Together, these studies show that sustained growth in bladder cancer models is not governed by a single variable. PDOs emphasize clonal competition and adaptation to drug pressure under in vitro conditions. BBN models emphasize damage accumulation and clonal selection during chronic carcinogen exposure. GEMMs reveal cooperation among proliferative signals, escape from damage responses, altered differentiation and microenvironmental pressure. Model optimization should therefore not simply aim to increase tumor take but should define which biological thresholds each model preserves and which thresholds it artificially bypasses. Only then can model results more accurately inform intravesical therapy, local delivery and large-animal translational studies.

Among these thresholds, adhesion is only the earliest step in local tumor establishment. Whether a small number of abnormal cells can move from transient survival to sustained expansion is often determined by their ability to adapt to oxidative stress, nutrient limitation, mitochondrial pressure and apoptotic signaling.

## 5. Metabolic Susceptibility: From Urothelial Homeostasis to Mitochondrial Stress Nodes

After adhesion and early retention, bladder cancer models must also explain how abnormal cells survive under local stress. The intravesical environment differs from subcutaneous tissue because it combines urine exposure, cyclic filling and emptying, inflammatory stimulation, oxidative stress and intravesical drug pressure. These conditions affect tumor cells and reshape host urothelial differentiation, repair and immune status. In this section, metabolic susceptibility refers to measurable metabolic or mitochondrial states that influence stress adaptation, apoptosis, immune interaction or treatment response. This definition keeps the discussion focused on variables that can be annotated or perturbed in models, rather than on cancer metabolism in general.

This logic is first apparent in normal urothelial homeostasis. The work of Liu and colleagues on PPARG showed that PPARG regulates not only urothelial differentiation, but also mitochondrial biogenesis, fatty-acid transport and resolution of inflammation after injury [84]. PPARG loss impaired maturation of superficial umbrella cells, promoted squamous-like differentiation of basal cells and prevented timely termination of nuclear factor-kappaB (NF-kappaB) activation after urinary tract infection, together with persistent inflammation, abnormal epithelial differentiation and basement membrane damage. These findings indicate that the urothelial barrier is not maintained solely by structural components such as UPs, tight junctions and the GAG layer. Its long-term stability also depends on metabolic transcriptional programs. Perturbation of these programs may shift the urothelium from a stable, differentiated and low-inflammatory barrier state towards a susceptible state marked by disordered repair, persistent inflammation and survival of abnormal cells.

In the tumor setting, PPARG-related studies further connect metabolic regulation with bladder cancer subtype, differentiation state and immune microenvironment. Tate and colleagues found that activation of PPARG signaling can drive basal-like cells towards a luminal-like state and, in the setting of BBN induction, generate bladder tumors resembling the luminal subtype. These tumors were enriched for metabolic and lipid-related pathways but showed lower T-cell activation, cytokine signaling and immune infiltration, consistent with an immune-cold phenotype [85]. Notably, some advanced luminal tumors gradually downregulated PPARG and other luminal markers and developed basal-like regions, suggesting that metabolic-differentiation programs can be remodeled during progression rather than remaining fixed. PPARG should therefore not be treated simply as an oncogenic or tumor-suppressive factor, but as a regulatory axis connecting urothelial differentiation, lipid metabolism, inflammatory restraint and tumor-subtype specification.

Proteogenomic evidence from patient samples supports this view. An integrated analysis of 448 samples from 190 patients with urothelial carcinoma across genomic, transcriptomic, proteomic and phosphoproteomic layers showed that papillary urothelial carcinoma (PUC) and CIS are not simple stages in a single linear progression pathway, but two progression branches with distinct molecular features. PUC was enriched for glycan and lipid metabolism, glycerophospholipid metabolism, glycolysis and gluconeogenesis, and was associated with lower immune infiltration [86]. CIS showed stronger DNA-damage response, apolipoprotein B messenger RNA (mRNA) editing enzyme, catalytic polypeptide-like (APOBEC)-related mutational signatures, immune signaling and EMT features. The study further suggested that PUC may rely more on metabolic reprogramming and an immune-cold local environment to sustain growth, whereas CIS resembles a high-damage, high-inflammation and high-invasion lesion state. Of particular note, glycolytic activity was negatively correlated with immune score and involved the enrichment of enzymes related to lactate production and secretion, indicating that metabolic activity does not necessarily increase immune recognition. Instead, metabolites such as lactate may contribute to formation of an immunosuppressive microenvironment. When invasive tumors were stratified by PUC-like or CIS-like protein features, PUC-derived invasive cancers were more luminal-like, whereas CIS-derived invasive cancers were closer to basal-squamous or neuronal features and were associated with poorer prognosis and higher metastatic risk [87].

Metabolic state is therefore not merely an accessory feature of tumor energy supply. It may help determine which early lesion gives rise to bladder cancer, which differentiation and immune trajectory the tumor follows and what invasive phenotype ultimately emerges. For model construction, this means that metabolic state should be treated as part of model identity and model susceptibility, rather than as a background variable interpreted only after drug-response experiments have been completed. This broader view is important because metabolic susceptibility can influence models through several routes. Some metabolic states shape lineage identity and immune exclusion, whereas others modify DNA-damage responses, interferon signaling, lactate-dependent immune suppression or mitochondrial apoptosis. The relevant question for model design is therefore not whether one pathway explains bladder cancer metabolism, but which metabolic state should be annotated, perturbed or controlled in a given model system.

Metabolic susceptibility also reshapes cell fate under treatment pressure. A study of the tryptophan metabolism-aryl hydrocarbon receptor (AhR)-stimulator of interferon genes (STING) axis showed that chemotherapy-induced type I interferon responses are an important component of anti-tumor activity, but tryptophan metabolites can activate AhR, promote STING degradation and suppress type I interferon (IFN-I) production, thereby weakening the effects of treatments such as cisplatin. Restricting tryptophan intake, blocking indoleamine 2,3-dioxygenase 1 (IDO1) or inhibiting AhR enhanced treatment response [88].

Work by Singh and colleagues on lysine demethylase 6A (KDM6A) further connected epigenetic alteration, metabolic reprogramming and treatment response within a single mechanistic chain. KDM6A is a chromatin regulator frequently mutated in bladder cancer. Its loss does not merely alter gene-expression profiles, but also reshapes DNA-damage repair, genome stability and metabolic state. The study showed that KDM6A loss weakens double-strand break repair and is accompanied by increased extrachromosomal circular DNA and resistance-associated amplification events, reducing tumor–cell sensitivity to DNA-damaging treatments such as cisplatin. KDM6A also helps maintain glycolysis in bladder cancer cells. After its loss, glycolytic and lactate-producing genes such as HK2, LDHA and PGAM1 are downregulated, intratumoral lactate accumulation decreases and oxidative phosphorylation-related features increase. Importantly, the study extended these metabolic changes to the immune microenvironment. Tumor-derived lactate normally supports the immunosuppressive function of regulatory T cells (Tregs) and shapes their transcriptional programs through histone lactylation. In KDM6A-deficient tumors, reduced lactate production decreases H3K9la and H3K18la in Tregs, lowers expression of immunosuppression-related genes such as Foxp3, Tgfb and Pdcd1, and restricts expansion of programmed cell death protein 1 (PD-1)-high Tregs [89]. As a result, PD-1 therapy increases the ratio of effector cluster of differentiated 8-positive (CD8+) T cells and Tregs and strengthens anti-tumor immunity. KDM6A loss therefore creates an apparently paradoxical but translationally informative therapeutic context: it promotes cisplatin resistance through defective DNA repair and genomic amplification, and increases sensitivity to immune-checkpoint blockade by weakening the lactate-Treg-lactylation axis.

Metabolic susceptibility should not be reduced to a single gene or transporter family. For model design, SLC25 transporters are best viewed as one operational entry point into mitochondrial stress, not as a complete organizing axis. Their value lies in their defined mitochondrial localization, measurable expression and direct links to metabolite exchange, redox balance, ROS generation and apoptotic threshold. This makes the SLC25 family useful for illustrating how metabolic variables could be incorporated into susceptibility-based model reporting.

The SLC25 family is located mainly in the mitochondrial inner membrane and transports a wide range of metabolites, including tricarboxylic-acid cycle intermediates, amino acids, nucleotides, fatty-acid-related substrates and cofactors. Unlike glycolytic enzymes or lipid-synthesis enzymes, SLC25 transporters sit at the interface between cytosolic and mitochondrial metabolism. Changes in their function can therefore simultaneously affect substrate supply, redox balance, ROS generation, mitochondrial membrane state and apoptotic threshold. This position makes the SLC25 family a useful route for moving from broad metabolic phenomena to operational molecular nodes of metabolic susceptibility.

Although bladder cancer studies have not yet produced a complete SLC25-based model system, several lines of evidence are informative. In normal urothelium, PPARG loss downregulates genes involved in fatty-acid transport and beta-oxidation, including Cpt1, Cpt2 and Slc25a20, suggesting that mitochondrial fatty-acid import is part of urothelial differentiation and metabolic homeostasis [84]. This observation is consistent with metabolomic studies in patients with bladder cancer. Kim and colleagues combined urinary metabolite profiling with tissue mRNA analysis and found perturbation of the carnitine-acylcarnitine pathway in patients with bladder cancer. Genes associated with fatty-acid entry into mitochondrial oxidation, including CPT1B, CPT1C, SLC25A20 and CRAT, were downregulated in NMIBC, suggesting that abnormal mitochondrial fatty-acid transport may already be present at an early disease stage [84,90]. These findings are consistent with the possibility that SLC25-related alterations appear during early metabolic remodeling of the urothelium. However, they do not yet establish a complete SLC25-based bladder cancer model. Future studies will need to test whether manipulating specific SLC25 transporters changes barrier state, colonization efficiency, oxidative stress tolerance or treatment response in orthotopic and carcinogen-induced settings.

SLC25A21 provides a more direct but still bounded example. Wang and colleagues reported that SLC25A21 showed tumor-suppressive properties in bladder cancer assays: overexpression inhibited proliferation, migration and invasion, promoted apoptosis and reduced xenograft growth. Mechanistically, SLC25A21 was linked to mitochondrial alpha-ketoglutarate transport, oxidative stress handling and ROS-mediated mitochondria-dependent apoptosis. These data support SLC25A21 as a candidate metabolic susceptibility node, but they do not yet show whether SLC25A21 manipulation changes orthotopic engraftment, BBN-induced progression or intravesical treatment response.

SLC25 members may also lie downstream of canonical oncogenic signaling. A study of ubiquitin C-terminal hydrolase L5 (UCHL5) showed that UCHL5 is upregulated in bladder cancer and promotes cell proliferation, migration and in vivo tumor growth through AKT/mTOR activation. RNA sequencing (RNA-seq) and protein validation further identified c-Myc, SLC25 member 19 (SLC25A19) and intercellular adhesion molecule 5 (ICAM5) as downstream altered molecules in this pathway [91]. This study did not by itself prove that SLC25A19 is required for bladder cancer progression, but it suggests that mitochondrial transport-related molecules can be incorporated into growth-promoting programs such as AKT/mTOR-c-Myc. SLC25 members may therefore function both as modulators of metabolic stress and apoptosis and as metabolic output nodes of tumor-growth signaling.

**Figure 3:**
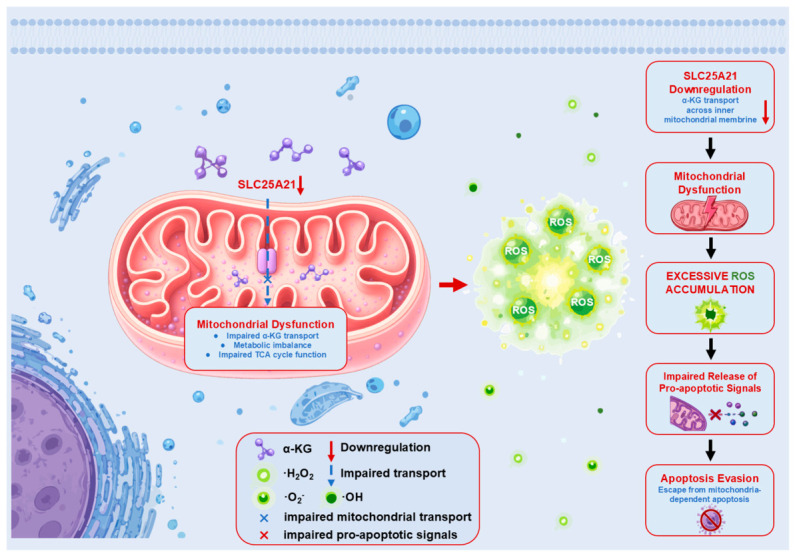
Metabolic susceptibility as a model-annotation layer in bladder cancer. PPARG-linked differentiation, PUC/CIS metabolic divergence, immune-metabolic regulation and mitochondrial stress nodes may influence tumor survival and treatment response. SLC25A21 is shown as an illustrative candidate linking mitochondrial transport, ROS and apoptosis, rather than as a validated model-defining axis.

Accordingly, SLC25A21 should be interpreted as an illustrative candidate node rather than as a validated model-defining axis. Direct evidence that SLC25A21 alters orthotopic engraftment, BBN-induced progression or intravesical treatment response remains limited, and these settings should be prioritized in future validation studies. This metabolic susceptibility framework is shown in Figure 3.

## 6. Translational Value of Large-Animal Susceptibility Models

The value of large-animal systems lies mainly in their translational scale, not in replacing small-animal mechanisms. Mouse models remain essential for causal testing of tumor initiation, pathway cooperation and immune context. However, they cannot fully reproduce repeated catheterization, device placement, cystoscopic monitoring, drug dwell, tissue penetration or local energy delivery at clinically relevant scale [92]. This section therefore treats rabbit, porcine and canine systems as platforms for intravesical delivery, imaging surveillance, device evaluation and selected naturally occurring diseases, while distinguishing these uses from de novo bladder carcinogenesis modeling.

For large-animal platforms to support translational claims, procedural and reporting variables should be defined before the model is interpreted. These variables include species or breed, body weight, bladder capacity or controlled filling volume, catheter route, instillation volume, intravesical pressure, dwell time, urine drainage, imaging schedule, device or energy settings, sampling sites, local toxicity and post-treatment bladder function. Such reporting is essential because the translational value of large-animal models lies not only in their anatomical scale but also in whether a treatment, imaging strategy or device can be delivered, monitored and assessed under clinically relevant bladder conditions.

The value of large-animal models lies in this translational space. They are not necessarily better than mice for dissecting mechanisms of tumor initiation, but they can cover aspects of intravesical therapy, local delivery, imaging surveillance and device feasibility that mouse models cannot. Rabbits provide one example. Functional studies have reported rabbit bladder capacities of approximately 47 mL, and some structural bladder models have reported capacities of about 34 mL [93]. These values vary with strain, body weight, anesthesia and experimental conditions and should not be treated as fixed physiological constants; however, they indicate that the rabbit bladder offers more room for catheter placement, local injection, intramural implantation and post-treatment pathological sampling. The VX2 rabbit bladder cancer model is built on this practical advantage. VX2 tumor tissue can be implanted into the rabbit bladder wall to generate rapidly growing bladder wall tumors with local invasion and central necrosis, and some models develop lymph-node or lung metastases [94]. This platform has been used to evaluate local treatments such as cryosurgery, high-energy shock waves and microwave coagulation, with pathological assessment of necrosis depth, local recurrence, extravesical extension and distant metastasis [95]. The rabbit VX2 model should therefore be positioned as an operable bladder wall tumor platform rather than a faithful model of natural human urothelial carcinogenesis. Its main value is procedural: it permits catheter-based access, intramural implantation, local therapy, imaging follow-up and pathological assessment of necrosis, recurrence and wall injury. These strengths make it useful for testing local treatment feasibility, but its xenogeneic origin and rapid growth limit claims about human tumor initiation or molecular subtype fidelity. For interpretation, rabbit VX2 studies should therefore report implantation site, tumor size at intervention, interval from implantation to treatment, imaging criteria for tumor establishment, necrosis depth, bladder wall injury, recurrence and metastatic assessment. These parameters help separate the procedural value of the model from claims about natural urothelial carcinogenesis, which VX2 tumors do not fully reproduce.

Pig models are more closely aligned with organ scale and device translation. Porcine bladders are often used to approximate human bladder wall structure, compliance and mechanical testing, making them useful for assessing instillation pressure, tissue deformation, local energy coverage and transurethral or percutaneous access routes. Some ex vivo porcine bladder perfusion models can maintain organ viability for several hours while permitting arterial-side drug delivery, intravesical drug administration, pressure recording and surface micromotion imaging [96]. These systems are well suited to comparing how different delivery routes affect bladder wall exposure and tissue response. In vivo porcine bladder cryoablation studies further show that different probes, freeze–thaw cycles and exposure times can generate relatively predictable full-thickness coagulative necrosis, and that cystoscopy, pathology and necropsy can be used to evaluate perforation, adjacent-organ injury and ablation extent [16]. These studies indicate that pig models are better positioned as preclinical platforms for safety and feasibility testing of intravesical devices, local ablation and delivery systems than as routine models of tumor mechanism. For porcine platforms, standardization should focus on organ mechanics, delivery route and device performance. Relevant parameters include filling volume, bladder compliance, catheter or cystoscopic access, perfusion route, instillation pressure, dwell time, tissue penetration, treated surface area, energy dose, ablation depth, perforation risk, adjacent-organ injury and post-procedural healing.

Canine models offer a different type of value. Experimental dogs or beagles can be used for bladder function monitoring, imaging assessment and validation of intravesical devices. Canine bladder capacity is often estimated at about 10 mL per kg body weight, and relevant studies can continuously record voided volume, intravesical pressure, abdominal pressure, detrusor pressure and urethral electromyographic activity. Three-dimensional ultrasonography has also been used to estimate canine bladder volume non-invasively [97,98]. These features make canine models suitable for assessing positioning, stability and safety of intravesical devices under realistic filling and emptying conditions. In addition, spontaneous invasive urothelial carcinoma in dogs provides a feature that rabbit and pig models cannot easily replace. Canine spontaneous bladder cancer shares aspects of histological behavior, local invasion, metastatic tendency and clinical management with human MIBC, and transcriptomic studies suggest luminal- and basal-like molecular features [99,100]. Because these tumors arise in immunocompetent hosts with genuine clinical heterogeneity, they can be used to evaluate targeted therapies, imaging tracers and systemic treatment response. In a folate receptor-targeted study, for example, nuclear imaging was first used to confirm folate uptake in primary or metastatic canine lesions, followed by dose escalation of the folate-vinblastine conjugate EC0905 [101]. The maximum tolerated weekly intravenous dose was approximately 0.25 mg per kg, with neutropenia and gastrointestinal toxicity as the main dose-limiting toxicities, and partial responses or stable disease were observed. Such data are closer to the translational questions that matter clinically than simple tumor formation: whether the drug reaches the lesion, whether the dose is tolerable and whether activity emerges in naturally heterogeneous tumors. For canine studies, the required reporting depends on whether the model uses healthy experimental dogs or spontaneous urothelial carcinoma. Functional or device studies should report bladder capacity, filling and voiding conditions, pressure recordings, device position, retention and mucosal injury. Studies of spontaneous canine tumors should additionally report tumor stage, anatomical location, prior treatment, imaging-defined tumor burden, molecular subtype when available, treatment dose, response criteria, toxicity and follow-up.

Large-animal models should not be viewed as enlarged mouse models. Rabbit models are particularly useful for bladder wall tumor implantation and local energy-based therapy. Pig models are better suited to organ mechanics, perfusion-based delivery and device-safety testing. Canine models have distinctive value in functional intravesical monitoring and spontaneous invasive tumors [99]. Together, these platforms compensate for the limited operative scale, imaging follow-up, intravesical therapy assessment and clinical workflow simulation of mouse models. They also have substantial boundaries, including high cost, limited sample size, difficult genetic manipulation, incomplete standardization and stricter ethical requirements. More importantly, except for spontaneous canine tumors, many large-animal studies still rely on normal bladders, injury models or xenogeneic implanted tumors and do not fully reflect the molecular subtypes and therapeutic heterogeneity of human bladder cancer [16,17].

A more realistic future direction is to treat large-animal platforms as the delivery and safety tier of a broader validation workflow. In such a workflow, patient-derived cells or PDOs first define molecular background and drug sensitivity; mouse orthotopic, BBN or GEMM systems then test mechanism and in vivo tumor behavior; and rabbit, porcine or canine platforms finally test whether the therapeutic route remains feasible under realistic bladder capacity, urinary dilution, catheter manipulation, tissue penetration and local toxicity constraints. This sequence avoids asking large animals to solve questions better suited to molecular or mouse models. PDOs and patient-derived cells can retain some histological features, mutational backgrounds and drug sensitivities of parental tumors and can distinguish biological contexts such as FGFR3 alteration, tumor protein p53 (TP53)/retinoblastoma 1 (RB1) deficiency, luminal–basal subtype, PPARG-related metabolic state and immune-cold or immune-hot phenotypes [2,85]. Large-animal models can then answer a different question: whether drugs, delivery systems or local therapeutic strategies that appear effective in vitro achieve sufficient local exposure under real bladder capacity, urinary dilution, cyclic emptying and bladder wall barrier conditions without causing unacceptable mucosal injury, wall necrosis or systemic toxicity. In this sense, patient-derived models define what the tumor is, whereas large-animal models test whether the treatment can reach and safely act on it.

This tiered strategy is particularly well suited to intravesical therapy research. In conventional mouse orthotopic models, drug dwell time, instillation pressure, catheter manipulation, bladder wall penetration depth and post-treatment bladder function are difficult to assess adequately. Combining molecularly annotated PDO screening with intravesical delivery, gel retention, nanoparticle penetration, photodynamic therapy, hyperthermia or cryoablation studies in rabbit or pig bladders would place tumor–cell killing and clinical executability within the same validation chain. For spontaneous canine bladder cancer, a further step is possible: development as a co-clinical research platform that integrates molecular subtyping, imaging tracers and response endpoints while retaining naturally arising tumors, immunocompetent hosts and real treatment responses. The goal of future model optimization is therefore not to replace mice with large animals, but to connect the mechanistic control of mouse models, the molecular authenticity of patient-derived models and the clinical operative scale of large-animal platforms into a multi-level system capable of evaluating barrier status, metabolic susceptibility, local delivery and treatment response. Platform-specific reporting would make these models more comparable and would prevent broad translational claims from being made on the basis of animal size or anatomy alone.

## 7. Validation Standards and Application Framework for Susceptibility-Engineered Models

As outlined in the Introduction, a susceptibility-engineered model cannot be judged by tumor take, tumor volume or growth inhibition alone. These endpoints remain useful, but they do not identify which constraints were preserved, intentionally modified or bypassed. We therefore propose that susceptibility-engineered bladder cancer models should be evaluated through four linked requirements: evidence of initiation or colonization threshold change, documentation of the bladder microenvironment, molecular interpretability and route-relevant therapeutic evaluation.

There are precedents for converting complex experimental systems into reportable, comparable and verifiable frameworks. The PDX-Minimal Information (PDX-MI) standard converts donor information, establishment procedure, passage history, host background, quality control and drug use into minimum reporting items for PDX models [85,102]. Animal Research: Reporting of In Vivo Experiments (ARRIVE) 2.0 and Planning Research and Experimental Procedures on Animals: Recommendations for Excellence (PREPARE) similarly emphasize transparency, reproducibility and methodological scrutiny in animal-experiment reporting and pre-experimental planning [103,104]. By analogy, susceptibility-engineered bladder cancer models cannot be judged adequately if tumor take or tumor volume is the only endpoint. This section therefore does not propose a fixed modeling protocol. Instead, it adapts this framework-based logic into a working evaluation scheme for bladder cancer model optimization to determine the following: whether susceptibility is established, whether the local process is observed, whether the molecular mechanism is interpretable and whether therapeutic outcomes have translational relevance.

To make this framework operational rather than purely descriptive, each susceptibility state should be linked to a defined variable, measurable readout, perturbation method, validation window and interpretation risk. This checklist, summarized in Table 2, is intended to distinguish susceptibility engineering from empirical model optimization, in which tumor take is increased without documenting which biological or translational constraint has been altered.

First, a model should demonstrate that it reflects tumor initiation or local colonization. In transplantation models, tumor formation is not a single endpoint after cell instillation, but a sequence that includes luminal contact, epithelial adhesion, resistance to urinary washout, early survival and local expansion. Erman and colleagues tracked the early behavior of the MB49 murine bladder cancer cell line in an orthotopic mouse bladder tumor model and found that tumor cells attached within a short time after intravesical instillation to the urothelium or basement membrane, using filopodia and local adhesion structures to establish contact with host tissue [106]. This suggests that the key issue in orthotopic transplantation is not only whether a tumor eventually forms, but whether exogenous cells actually experience the intravesical colonization step. Accordingly, if a barrier intervention or pretreatment is claimed to create susceptibility, early time-point imaging, fluorescent tracing, histology and markers of apoptosis and proliferation should be used to show that adhesion, retention and expansion have changed, rather than simply documenting a larger tumor at the endpoint [107]. For BBN and GEMMs, validation should focus on whether damage accumulation, clonal expansion and invasive progression are faithfully captured. Kobayashi and colleagues noted that many genetic alterations produce only hyperplasia, dysplasia or non-invasive lesions, whereas entry into muscle-invasive or metastatic disease often requires specific pathway combinations. Matye and colleagues likewise emphasized that strain, dose, exposure duration and observation window shape the lesion spectrum in BBN models. Non-transplant models should therefore not merely report whether tumors arise, but should show whether lesions follow an interpretable temporal sequence from injury to hyperplasia, CIS or papillary disease and then invasion.

Second, except for in vitro cell-line and organoid models used for mechanism validation or selected pharmacological studies, animal models should demonstrate preservation of the bladder microenvironment. Bladder cancer is distinctive because the tumor does not arise in generic soft tissue, but within a local niche consisting of the urothelial barrier, urine exposure, cyclic filling and emptying, basement membrane, lamina propria, immune cells and stroma [108]. Subcutaneous CDX or PDX models are convenient for measuring tumor volume and systemic drug response, but their tumor-forming process bypasses the urothelial barrier and intravesical pressure, and they cannot replace orthotopic models for evaluating local colonization or intravesical therapy [92]. Conversely, orthotopic models are closer to the bladder environment but often rely on acid-base treatment, mechanical injury, electrocautery or poly-L-lysine to increase adhesion [32]. These procedures introduce wound repair, inflammation and stromal exposure as additional variables. A rigorous susceptibility model should therefore do more than show improved engraftment after treatment. It should assess UPK expression, the GAG layer, tight junctions, basement membrane integrity, inflammatory mediators, immune cell infiltration and stromal remodeling to determine whether the model has gently opened a colonization window or instead converted the bladder into a wound-repair environment. Only when local structure and inflammatory status are documented does increased tumor take become interpretable.

Third, the model should have molecular interpretability. With The Cancer Genome Atlas and international consensus classification, bladder cancer is no longer viewed as a single disease, but as a set of molecular subtypes, including luminal, basal-squamous, stroma-rich and neuroendocrine-like states, each with distinct differentiation, immune and therapeutic associations [2]. The MIBC consensus classification proposed by Kamoun and colleagues provides a reference for model annotation, and the multi-omics analysis by Yao and colleagues of 448 samples from 190 patients further suggests that papillary urothelial carcinoma and CIS represent progression branches with different metabolic, DNA-damage and immune features [87]. Future models should therefore not merely claim higher tumor take, but should specify which patient context the model resembles. Recent targeted next-generation sequencing of paired tumor and blood samples from patients with urothelial bladder carcinoma identified recurrent somatic alterations in FGFR3, TSC1, PIK3CA and TP53, and linked FGFR3 and TP53 variants with tumor invasiveness, grade or stage, supporting the use of clinically anchored genomic alterations as model-annotation variables [109]. For example, BBN models can show basal-subtype-like expression and alterations in epigenetic regulators such as Kmt2c and Kmt2d. Tate and colleagues showed that PPARG signaling can shape bladder cancer differentiation state and immune-exclusion features [85]. These findings indicate that model susceptibility is not a purely morphological concept. It should be definable by molecular markers, transcriptomes, proteomes or metabolic states. If a model claims to alter tumor progression through barrier or metabolic susceptibility, it should further specify whether this is accompanied by changes in luminal–basal subtype, FGFR3 alteration, TP53/RB1 deficiency, PPARG activity, glycan or lipid metabolism, mitochondrial stress or immune-cold versus immune-hot status.

Finally, the model should serve therapeutic evaluation rather than modeling success alone. Different models answer different therapeutic questions. Cell lines are useful for rapid screening of cell-intrinsic drug sensitivity, whereas PDOs better preserve the histological and molecular heterogeneity of patient-derived tumors. The bladder cancer PDO models established by Lee and colleagues retained aspects of parental-tumor heterogeneity and were used to study tumor evolution and drug response. Minoli and colleagues further used PDOs from bladder cancers of different stages and grades to assess drug response, supporting the role of PDOs as a bridge between molecular subtyping and functional screening. For intravesical therapy, local delivery, drug dwell and bladder wall penetration, however, in vitro sensitivity is not enough. The transurethral orthotopic bladder cancer model combined with bioluminescence imaging developed by Hadaschik and their colleagues highlights the need for longitudinal monitoring of tumor burden and evaluation of intravesical therapy. Large-animal models further enable assessment of instillation pressure, catheter manipulation, imaging follow-up, local toxicity and device safety. Therapeutic evaluation is therefore not a search for a universal model, but a problem-based combination of PDOs, mouse orthotopic models, BBN/GEMMs and large-animal platforms. In practical terms, patient-derived models can first define molecular background and drug sensitivity, mouse models can then test mechanism and in vivo growth, and large-animal bladder platforms can finally assess delivery, retention, penetration and safety.

A susceptibility-engineered bladder cancer model should meet four minimum evidentiary requirements. First, it should show whether the proposed susceptibility state changes initiation, adhesion, retention, survival or local expansion. Second, it should document the bladder microenvironment, including barrier integrity, basement membrane exposure, inflammatory status, immune infiltration or stromal remodeling when relevant. Third, it should define molecular identity through subtype markers, genomic alterations, transcriptomic/proteomic features or metabolic readouts. Fourth, therapeutic testing should match the intended route and clinical question, for example systemic drug response, intravesical dwell, wall penetration, device safety or local toxicity. If an intervention increases tumor take without linking these levels, it should be described as empirical model optimization rather than susceptibility engineering.

Together, these four requirements form a tiered validation chain that links molecular definition, mechanistic testing and local translational evaluation. This tiered validation chain is summarized in Table 3.

## 8. Conclusions and Future Perspectives

Bladder cancer models should be judged by the constraints they preserve, not only by tumor take, growth rate or convenience. This review proposes susceptibility engineering as a model selection and validation logic that links barrier integrity, colonization thresholds, metabolic state and translational scale. Cell lines, PDOs, CDXs, PDXs, orthotopic transplantation, BBN-induced tumors, GEMMs and large-animal platforms each preserve different parts of this chain. Their value therefore depends on the question being asked, the susceptibility state being tested, and the readouts used to verify it.

The urothelial barrier and metabolic state are two informative susceptibility variables. UPs, the AUM, the GAG layer and tight junctions create a low-adhesion luminal surface that constrains orthotopic engraftment. PPARG-associated differentiation programs and PUC/CIS metabolic differences support the inclusion of metabolic state in model annotation. SLC25-linked mitochondrial transport, including SLC25A21, provides an illustrative candidate route for connecting metabolic stress, ROS and apoptosis to model susceptibility. However, this axis requires further validation in orthotopic, carcinogen-induced and treatment-response settings.

Future model optimization should translate these variables into reportable standards covering colonization, microenvironment, molecular identity and therapeutic route. Rather than expecting one system to answer every question, tiered platforms should connect patient-derived molecular authenticity, mouse-based mechanistic control and the operative scale of rabbit, porcine or canine models.

Susceptibility engineering should not mean weakening the host barrier simply to increase tumor take. Excessive mechanical or chemical injury may shift a model towards wound repair and inflammation, reducing its value for authentic bladder carcinogenesis. Metabolic nodes such as SLC25A21 and large-animal platforms also remain evidence-building areas, and their causal roles, reproducibility and cross-model applicability require further validation.

The next step is to build model chains that explain why tumors form, progress and respond to therapy under defined local constraints. Integrating barrier readouts, colonization thresholds, metabolic annotation and operative scale could turn model comparison into a more mechanistic design strategy. As gene editing, PDOs, multi-omics annotation and large-animal local-therapy platforms mature, the most useful bladder cancer models will likely be stratified by the biological constraint they preserve and by the clinical decision they are designed to support.

## Figures and Tables

**Table 1:** Major bladder cancer model platforms capture different susceptibility constraints and are therefore best matched to distinct mechanistic, pharmacological and translational questions.

Model Platform	Core Strengths	Main Limitations	Susceptibility Dimensions Represented	Best-Fit Applications	Reference
2D cell lines	Fast, scalable and experimentally controllable	No tissue architecture, urine exposure, barrier selection or immune-stromal context	Cell-intrinsic growth; pathway dependence; drug sensitivity	Target validation; mechanistic screening; high-throughput pharmacology	[57]
PDOs and patient-derived cells	Preserve patient heterogeneity and enable subtype-linked testing	Limited immune, stromal, vascular and bladder-lumen context	Clonal fitness; drug pressure; metabolic adaptation	Patient stratification; functional drug prioritization; bridge to in vivo validation	[20]
CDX and PDX models	Support in vivo tumor growth; PDXs retain patient-derived features	Subcutaneous models bypass the bladder niche; frequent immunodeficiency	Tumor expansion; vascular support; systemic drug response; resistance evolution	Preclinical efficacy; resistance studies; molecularly guided therapy	[58]
Orthotopic bladder transplantation	Preserves bladder-site growth and intravesical access	Engraftment depends on barrier injury and pretreatment intensity	Luminal contact; adhesion; urinary washout resistance; local expansion	Colonization studies; intravesical therapy; local drug delivery	[32]
BBN-induced chemical carcinogenesis	Models de novo tumor initiation in immunocompetent hosts	Long latency; pathology varies by strain, sex, dose and duration	Carcinogen exposure; epithelial injury; inflammation; clonal selection	Initiation and progression mechanisms; chemoprevention; immune studies	[9]
GEMMs	Test defined genetic drivers in native urothelium	Few models reproducibly generate invasive or metastatic disease	Driver cooperation; lineage state; differentiation; progression thresholds	Causal pathway testing; subtype biology; genetic interaction studies	[59]
Large-animal bladder platforms	Provide clinical scale for catheter, imaging, device and local therapy testing	High cost; low throughput; limited genetics; incomplete molecular matching	Dwell time; urine dilution; wall penetration; local toxicity; procedural feasibility	Intravesical delivery; device validation; imaging; local ablation; co-clinical studies	[16]

**Table 2:** The checklist defines how major susceptibility domains can be measured, perturbed and interpreted across bladder cancer model systems. It is intended to separate biologically defined susceptibility from empirical increases in tumor take or growth. The listed examples are representative rather than exhaustive.

Susceptibility Domain	Operational Readouts	Perturbation Strategy	Validation Window	Main Interpretive Risk	Reference
Urothelial barrier	UPKs, GAG layer, tight junctions, injury score	Barrier injury or modulation	Pre-implantation to early engraftment	Wound repair mistaken for susceptibility	[60]
Adhesion and retention	Labelled-cell retention, washout resistance, early apoptosis	Cell dose, dwell time, mucosal priming	Hours–days	Endpoint burden obscures failed attachment	[105]
Local expansion	Ki-67, apoptosis, invasion depth, serial imaging	Orthotopic grafting, BBN or GEMM combinations	Days–months; model-dependent duration	Growth reflects bypassed native selection	[9]
Bladder microenvironment	Immune cells, cytokines, stroma, basement membrane	Injury level, strain, immune background	Baseline, early response, endpoint	Inflammation confounds tumor phenotype	[106]
Molecular identity	Subtype markers, FGFR3, TP53/RB1, PPARG, omics	Patient-derived selection or pathway perturbation	Baseline and endpoint	Anatomical fidelity masks molecular mismatch	[87]
Metabolic susceptibility	ROS, mitochondrial activity, lactate, apoptosis, SLC25 markers	PPARG/SLC25 modulation, metabolic or drug stress	Baseline, stress phase, treatment phase	Association mistaken for causality	[88]
Intravesical exposure	Dwell time, wall penetration, imaging, local toxicity	Formulation, device, instillation volume, large-animal testing	Delivery–follow-up	In vitro efficacy fails under bladder-scale constraints	[16]

**Table 3:** A tiered validation chain for susceptibility-engineered bladder cancer models. Patient-derived systems define molecular context, small-animal models test mechanism and local tumor behavior, and large-animal platforms evaluate route-specific delivery, imaging, device safety and local toxicity.

Tier	Platform	Primary Role	Core Readouts	Decision Supported	Reference
1. Molecular definition	Patient tissue; urine-derived samples; subtype annotation	Anchor the model to a defined disease context	Histology; mutations; transcriptome/proteome; metabolic markers	Patient context for interpretation	[2]
2. Functional screening	PDOs; patient-derived cells; short-term cultures	Prioritize intrinsic drug sensitivity or resistance	Viability; IC50; apoptosis; organoid morphology	Candidate drugs or combinations	[20]
3. Mechanistic validation	Orthotopic CDX/PDX/PDO xenografts; BBN models; GEMMs	Test initiation, colonization, progression and resistance mechanisms	Adhesion; tumor take; imaging; histology; immune infiltration; UPK/GAG/tight-junction status	Causal in vivo mechanism	[8,9]
4. Local translational testing	Rabbit, pig and canine bladder platforms	Evaluate delivery, retention, monitoring and local safety	Cystoscopy; penetration depth; device feasibility; mucosal injury; systemic toxicity	Clinical feasibility of intravesical therapy	[16,99]
5. Integrated interpretation	PDO to mouse to large-animal validation chain	Align molecular identity, mechanism and delivery feasibility	Cross-model efficacy; exposure; safety; subtype-specific response	Rational model selection for translation	[20]

## Data Availability

No new data were created or analyzed in this study. Data sharing is not applicable to this article.

## Abbreviations

2D	two-dimensional
Ad5	adenovirus type 5
Adeno-Cre	adenovirus expressing Cre recombinase
AhR	aryl hydrocarbon receptor
AKT	protein kinase B
ARRIVE	Animal Research: Reporting of In Vivo Experiments
AR	androgen receptor
AUM	asymmetric unit membrane
BALB/c	BALB/c mouse strain
BBN	N-butyl-N-(4-hydroxybutyl) nitrosamine
C57BL/6	C57BL/6 mouse strain
CAR	coxsackievirus and adenovirus receptor
Cas9	CRISPR-associated protein 9
CD8	cluster of differentiation 8
CDX/CDXs	cell-line-derived xenograft(s)
CG8840	urothelium-specific oncolytic adenovirus CG8840
CIS	carcinoma in situ
CK19	cytokeratin 19
COXEN	co-expression extrapolation
CPT1B	carnitine palmitoyltransferase 1B
CPT1C	carnitine palmitoyltransferase 1C
Cpt1	carnitine palmitoyltransferase 1
Cpt2	carnitine palmitoyltransferase 2
CRAT	carnitine O-acetyltransferase
CRISPR	clustered regularly interspaced short palindromic repeats
CRISPR/Cas9	clustered regularly interspaced short palindromic repeats/CRISPR-associated protein 9
DNA	deoxyribonucleic acid
E1A	adenoviral early region 1A
E1A-AR	adenoviral early region 1A-androgen receptor fusion gene
E2F1	E2F transcription factor 1
EC0905	folate-vinblastine conjugate EC0905
EGFR	epidermal growth factor receptor
EGFP	enhanced green fluorescent protein
EMT	epithelial–mesenchymal transition
F344	Fischer 344 rat strain
FGFR	fibroblast growth factor receptor
FGFR3	fibroblast growth factor receptor 3
FGFR3-TACC3	fibroblast growth factor receptor 3-transforming acidic coiled-coil containing protein 3 fusion
FOXA1	Forkhead box A1
Foxp3	Forkhead box P3
FVB	FVB mouse strain
GAG	glycosaminoglycan
GATA3	GATA binding protein 3
GEMM/GEMMs	genetically engineered mouse model(s)
H3K9la	histone H3 lysine 9 lactylation
H3K18la	histone H3 lysine 18 lactylation
HER2	human epidermal growth factor receptor 2
HES1	Hes family bHLH transcription factor 1
HK2	hexokinase 2
H-Ras/HRAS	Harvey rat sarcoma viral oncogene homolog
Hras/Kras	Harvey rat sarcoma/Kirsten rat sarcoma viral oncogene homolog
HSV-TK	herpes simplex virus thymidine kinase
IC50	half-maximal inhibitory concentration
ICAM5	intercellular adhesion molecule 5
ICR	Institute of Cancer Research mouse strain
IDO1	indoleamine 2,3-dioxygenase 1
IFN-I	type I interferon
KDM6A	lysine demethylase 6A
KRT20	keratin 20
KRT5/14	keratin 5/14
Kmt2c	lysine methyltransferase 2C
Kmt2d	lysine methyltransferase 2D
LacZ	beta-galactosidase reporter gene
LDHA	lactate dehydrogenase A
MAPK	mitogen-activated protein kinase
MB49	MB49 murine bladder cancer cell line
MEK	mitogen-activated protein kinase kinase
MIBC	muscle-invasive bladder cancer
mRNA	messenger RNA
mTOR	mechanistic target of rapamycin
NF-kappaB	nuclear factor kappa B
NMIBC	non-muscle-invasive bladder cancer
NON/Shi	NON/Shi mouse strain
NOTCH1	notch receptor 1
NOTCH2	notch receptor 2
p21	cyclin-dependent kinase inhibitor 1A
p53	tumor protein p53
p63	tumor protein p63
p107	retinoblastoma-like protein 1
PD-1	programmed cell death protein 1
PDO/PDO(s)	patient-derived organoid(s)
PDX/PDX(s)	patient-derived xenograft(s)
PDX-MI	atient-derived xenograft-minimal information
PGAM1	phosphoglycerate mutase 1
PI3K	phosphoinositide 3-kinase
PPARG/PPARG	peroxisome proliferator-activated receptor gamma
PREPARE	Planning Research and Experimental Procedures on Animals: Recommendations for Excellence
PSCAE	prostate stem cell antigen enhancer
PTEN	phosphatase and tensin homolog
PUC	papillary urothelial carcinoma
RAS	rat sarcoma viral oncogene homolog
RAS/MAPK	rat sarcoma/mitogen-activated protein kinase pathway
Rb	retinoblastoma protein
Rb1	retinoblastoma 1
Rbpj	recombination signal binding protein for immunoglobulin kappa J region
RNA-seq	RNA sequencing
ROS	reactive oxygen species
rAAV9	recombinant adeno-associated virus serotype 9
rAAV9-UPII-TK-EGFP	recombinant adeno-associated virus serotype 9-uroplakin II-thymidine kinase-enhanced green fluorescent protein
siRNA	small interfering RNA
SLC25	solute carrier family 25
SLC25A19	solute carrier family 25 member 19
SLC25A20/Slc25a20	solute carrier family 25 member 20
SLC25A21	solute carrier family 25 member 21
STING	stimulator of interferon genes
SV40	simian virus 40
Stag2	stromal antigen 2
T24	T24 human bladder cancer cell line
TACC3	transforming acidic coiled-coil containing protein 3
Tgfb	transforming growth factor beta
TK	thymidine kinase
TP53	tumor protein p53 gene
TP53/RB1	tumor protein p53/retinoblastoma 1
Treg/Tregs	regulatory T cell(s)
Trp53	transformation-related protein 53
UCHL5	ubiquitin C-terminal hydrolase L5
UP/UPs	uroplakin(s)
UPII	uroplakin II
UPK	uroplakin
UPK1A	uroplakin 1A
UPK1B	uroplakin 1B
UPK2	uroplakin 2
UPK3A	uroplakin 3A
UPK3B	uroplakin 3B
VX2	VX2 tumor model
Wnt	wingless-related integration site signaling pathway
Wnt/beta-catenin	wingless-related integration site/beta-catenin signaling pathway
